# Caries prevalence and other dental pathological conditions in Vikings from Varnhem, Sweden

**DOI:** 10.1371/journal.pone.0295282

**Published:** 2023-12-13

**Authors:** Carolina Bertilsson, Maria Vretemark, Henrik Lund, Peter Lingström

**Affiliations:** 1 Department of Cariology, Institute of Odontology, Sahlgrenska Academy, University of Gothenburg, Gothenburg, Sweden; 2 Västergötlands Museum, Skara, Sweden; 3 Department of Oral & Maxillofacial Radiology, Institute of Odontology, Sahlgrenska Academy, University of Gothenburg, Gothenburg, Sweden; Vilnius University: Vilniaus Universitetas, LITHUANIA

## Abstract

In a late Swedish Viking Age population dating from around 10^th^-12^th^ century AD, the prevalence, distribution and location of dental caries were studied. Tooth wear, other dental pathology and anatomical variations were identified and recorded clinically and radiographically. A total of 3293 teeth were analyzed from 171 individuals with complete and partial dentitions, of which 133 were permanent and 38 deciduous/mixed dentition. The dentitions were studied clinically, using a dental probe under a strong light source, and radiographs were taken for 18 of the individuals to verify and complement the clinical caries registration. Almost half the population, 83 of 171 individuals (49%), had at least one carious lesion. All individuals with deciduous or mixed dentitions were caries-free. The number of teeth affected by caries among adults was 424 (13%) and the surface most susceptible to caries was the root surface. The tooth most commonly affected by caries was the first mandibular molar. Other findings included apical infections, which were detected clinically in 4% of the teeth, and one case of filed front teeth. The findings gave a unique understanding of life and death in this early Christian Viking community and indicated that it was common to suffer from dental caries, tooth loss, infections of dental origin and tooth pain. These Vikings also manipulated their teeth through filing, tooth picking and other occupational behaviors.

## Introduction

In 2005, excavations behind Varnhem Abbey, in the municipality of Skara, Sweden, revealed the ruins of a Christian stone church built in the early 11th century. To date, this is the oldest known stone church in Sweden (excluding the Scania area which was part of the Danish kingdom at this time) and the stone church was preceded by an even older wooden church. In close proximity to the church, an extensive cemetery comprising of thousands of graves dating from the 10^th^-12^th^ century was identified. Of these, a total of 300 have been undergone archaeological and osteological analysis [1].

The jaws and teeth in the Viking Age remains excavated near the Varnhem stone church can provide valuable evidence of everyday life in this unique early Christian settlement, since these highly mineralized structures are well-preserved post mortem [2]. Findings from osteoarchaeological dental examinations relate to general health and disease, nutritional composition and frequency, perceived pain and oral discomfort and everyday tasks. Findings such as dental caries, infections of the jaws, and dental wear provide a unique understanding of life in historical times [2]. Thorough examinations of tooth tissues such as enamel, dentine and dental pulp, can provide answers relating to microbiota [3, 4], nutritional isotopes [5–7] and heritage [8], which can enhance our knowledge of ancient populations.

In the literature, a number of studies conclusively indicate an increasing prevalence of dental caries as time progressed from prehistoric times to the 19th century [9], with a peak during the early and mid-20th century, due to the introduction of highly processed food stuffs and the increasing availability of white sugar [10–18]. To date, only a small amount of published scientific works regarding the dental health of Viking remains in Sweden exist, however, a case report from the Swedish island of Gotland states a high prevalence of dental caries, tooth wear and infections in the alveolar bone [19]. Meanwhile, a study of Icelandic Vikings found “only a few carious lesions, but a high degree of tooth wear” [20]. In an osteoarcheological study of Danish Viking remains, dental caries were found to have affected approximately 30% of the individuals and 3–5% of the teeth [21], while the dentitions displayed advanced wear, as well as a large number of infections in the jaws. Lunt [22] has reported caries rates of approximately 3% of the teeth in a Scottish Viking Age settlement. The unique and extensive collection of remains from the Varnhem settlement can provide a comprehensive idea of oral health in Sweden during the Viking Age. Previous research into the nutrition and mobility of the Varnhem Vikings concluded that the population was largely non-local with a great deal of mobility and that their diet was based mainly on terrestrial resources with smaller amounts of aquatic resources [23].

The aim of this study was to study dental caries and other oral health-related conditions in dentitions from the Viking Age from Varnhem, Sweden.

## Materials and methods

### Study population

Of the 300 individuals previously examined by archaeologist at Västergötlands museum, a total of 171 partial and complete dentitions were suitable for dental examination. The inclusion criterion for dental examination was the presence of complete or fragmented maxillae, mandibulae or both and selection was made by osteologist MV. Exclusion criteria was lack of complete or fragmented maxillae, mandibulae or both. Age and sex had already been estimated by the same osteologist before dental examinations according to Buikstra & Ubelaker [24]. The study population was divided into two groups based on the presence of permanent or deciduous/mixed dentition. Individuals with an estimated age above 12 years with single persisting deciduous tooth were placed in the permanent dentition group. The group with permanent dentition consisted of 133 individuals, while the group with deciduous and mixed dentition comprised of 38 individuals. Of the 133 individuals with permanent teeth, 46 were female and 87 were male. Derived from the median of the estimated age range at death for each individual, the mean age at death in this group was 35 years old, and the age at death ranged from 14 to over 50 years old. In the group with deciduous or mixed dentition, age at death raged from <1 year to 12 years old.

### Clinical examination

Clinical examinations were performed under a strong light source, using a dental probe. The examination of teeth and jaws was performed by a dentist (CB) and two undergraduate dental students. The students each examined half the material, while the dentist examined all the dentitions a second time. Inter-examiner tests were carried out at the beginning of every day of examination and a comparison between students and the dentist was made at the end of the examination phase. In the event of disagreement, a dentition was studied a second time.

The number of teeth (remaining and lost), carious lesions, tooth wear, apical pathology, and other findings of interest were registered. The alveolar bone was examined to determine whether a missing tooth was lost post- or ante-mortem. Empty alveolar sockets without signs of healing indicated teeth lost post-mortem. Root remains were recorded as remaining teeth, but, if the crown of a tooth was fractured post-mortem, this tooth was recorded as lost post-mortem.

Only cavitated manifest lesions which could be explored with a dental probe on the crown or root of teeth were determined to be carious. Superficial discolorations were not determined as carious, since the long period that had passed since burial and the conditions of the burial and surrounding soil could easily have affected the appearance of the enamel. The location of recorded lesions was classified as one or more of the following: 1) buccal, 2) distal, 3) lingual/palatal, 4) mesial, 5) occlusal and/or 6) root caries. The individual caries status was indicated by DMT index summarizing the number of decayed (D) and missed (M) teeth.

The severity of dental wear was scored using the system suggested by Johansson et al. [25] from 0 to 4: 0 = no visible facets in the enamel, 1 = marked wear facets in the enamel, 2 = wear into the dentine, 3 = extensive wear into the dentine and 4 = wear into the secondary dentine. This method was used to register wear on the first incisors and first molars in the lower jaw. If the first incisor and/or molar was present in both quadrants, the registration was performed on the one with the greatest degree of tooth wear. In cases where these teeth were missing, no registration was made. Only permanent teeth were included in this registration. As a result, if only deciduous teeth were present, no registration was made.

### Radiographic examination

In order to verify and complement the clinical caries registration, 18 individuals were randomly chosen for a radiographic examination using the bitewing technique. The selection was made by one of the authors (CB) based on the following inclusion criterion–several remaining posterior teeth in the alveolar bone in conjunction with each other, with intact approximal contact surfaces. The bitewing examination was performed at right angles to the teeth, parallel to the arches, to ensure that interproximal spaces and contact surfaces, as well as the interproximal bone, were clearly depicted. The radiographic examination was performed using a PSP (Photostimulable Phosphor Plate) detector and a Digora^TM^ Optime scanner (Soredex^TM^, KaVo Scandinavia AB, Sollentuna). The exposure parameters that were used were 75 kV and 0.250 s. After examination, each image was exported to the tiff format (16-bit grayscale) for subsequent viewing and interpretation on a high-resolution computer screen. CB performed the diagnostics and recorded the carious lesions, which were only included as carious sites when they presented a clear advancement into the dentine (manifest caries). Further, any incidental findings on the radiographs were recorded.

### Statistical work

Statistical work was performed using the SPSS programme software and the tests used included chi-square and Pearson’s correlation test. Significance was set at p < 0.05.

### Additional information

The conduction of current archaeological examination has been approved by the county administrative board (Länsstyrelsen Västra Götaland). No additional permits were obtained for the prescribed study which complied with all relevant regulations. Location of the studied remains is Västergötlands Museum, Stadsträdgården, Skara, Sweden.

### Ethics statement

Ethical approval was not applicable for this study due to the Swedish laws and regulations regarding archaeological remains. The conduction of current archaeological examination has been approved by the county administrative board (Länsstyrelsen Västra Götaland). No further ethical approval is applicable since the odontological examination of these archeological remains was part of the osteological (archaeological) examination which was undertaken by Västergötlands museum (institute in charge). All examinations were carried out in accordance to relevant guidelines.

## Results

### Lost teeth

**Fig 1:**
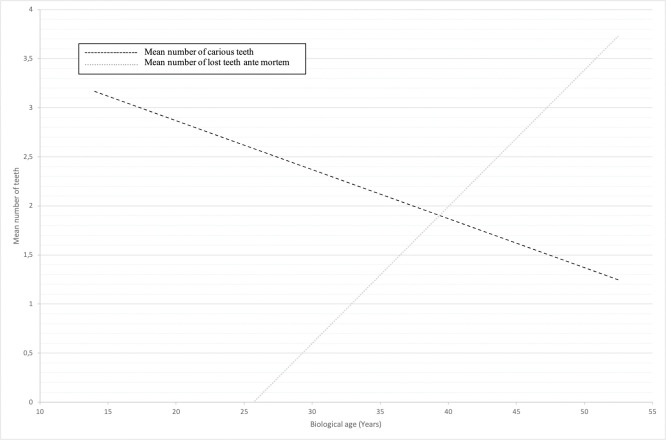
Correlation between lost and carious teeth.

The total number of examined individuals with permanent dentitions was 133 and the number of examined teeth (including wisdom teeth) was 3,293. The total number of lost teeth was 953 (23%), of which 219 were wisdom teeth. Of the 744 (20%) teeth (not including wisdom teeth) lost, 26% were lost ante mortem (AM) and 74% were lost post mortem (PM). In total, 6% of the teeth (excluding the wisdom teeth) in the adult population was lost ante mortem. A positive correlation (p<0.05) was found for teeth lost ante mortem and age (see Fig 1).

### Caries

**Fig 2:**
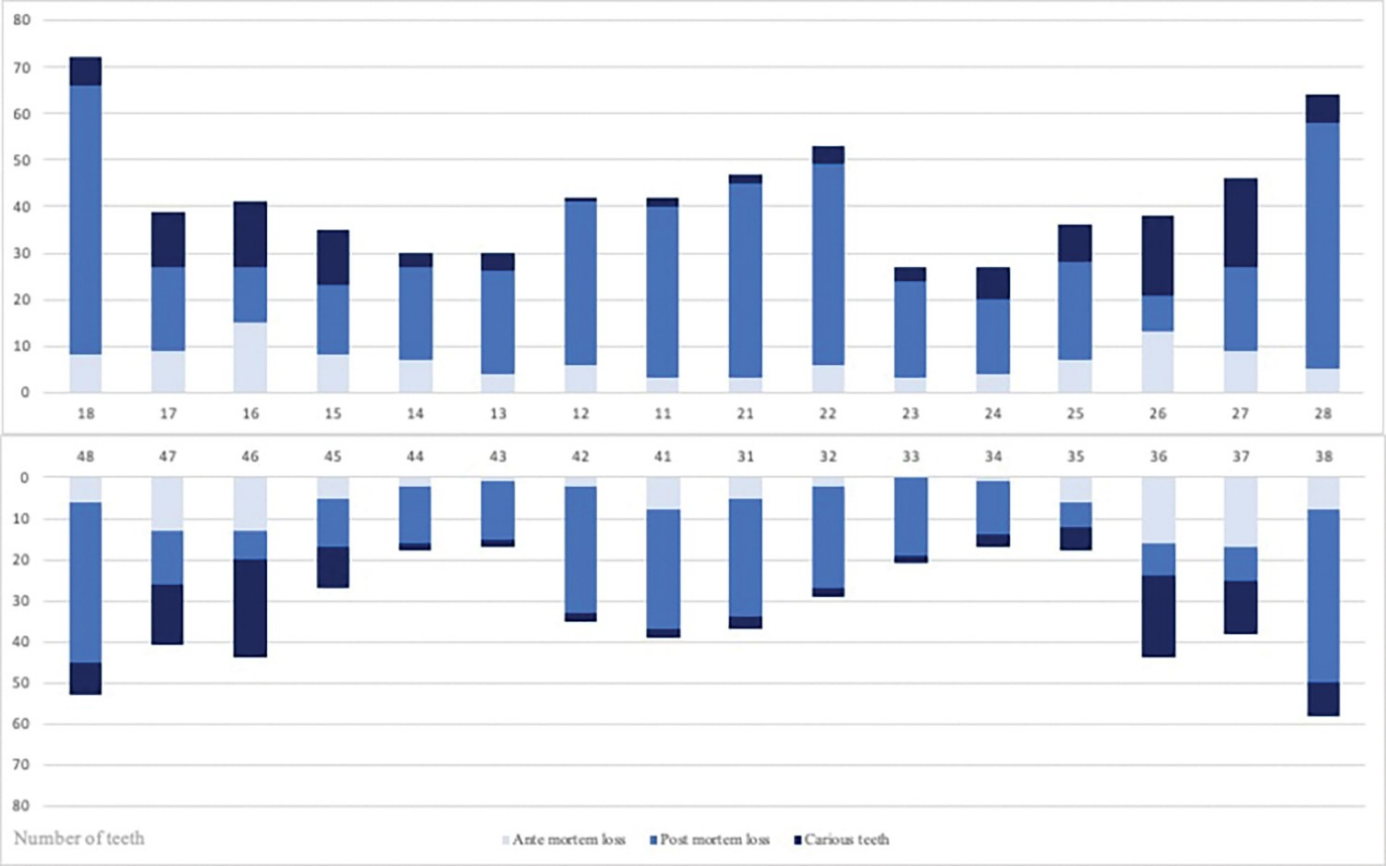
Number of teeth with caries, and lost ante and post mortem, according to tooth number.

**Fig 3:**
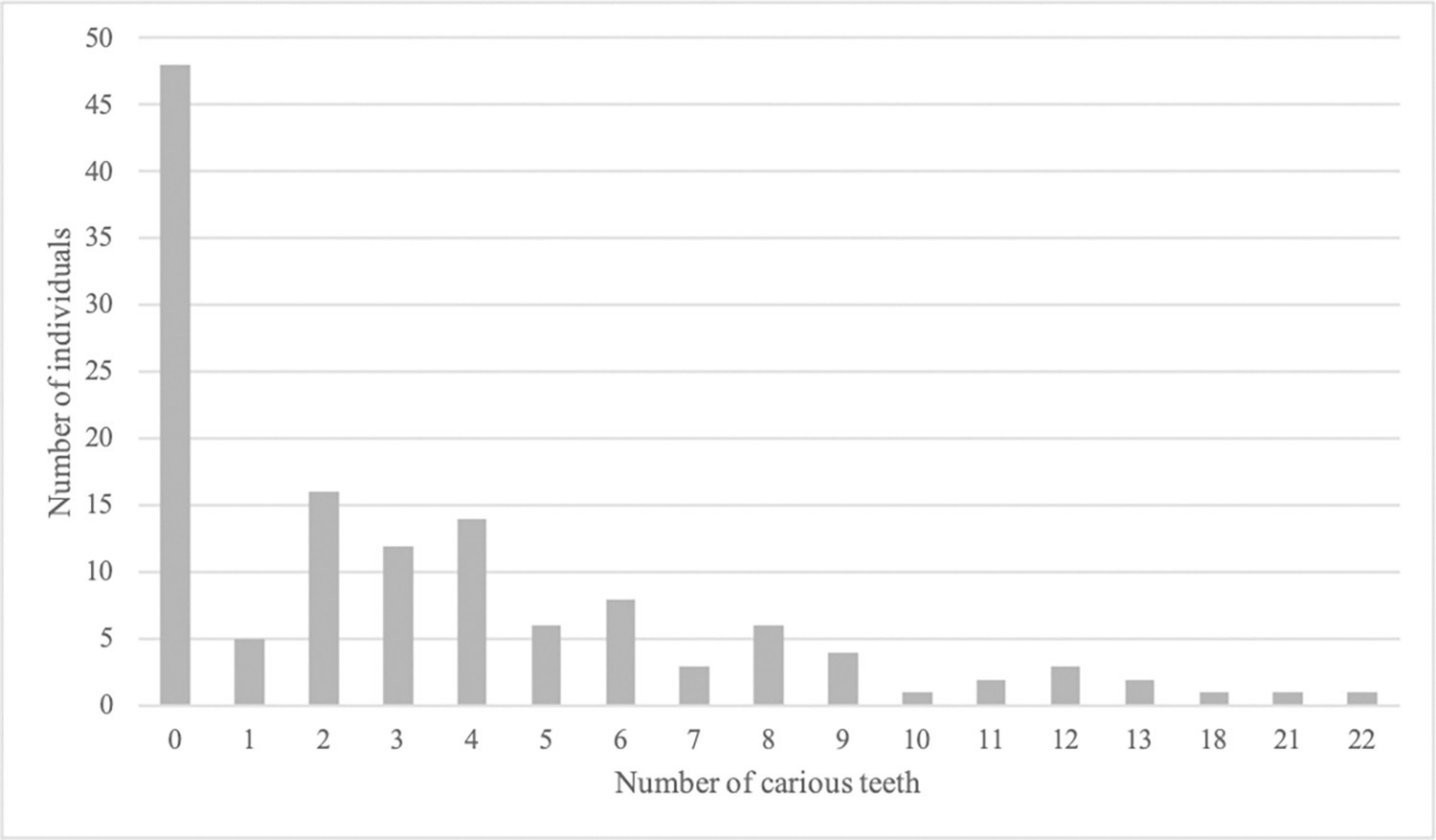
Number of carious teeth per individual.

**Fig 4:**
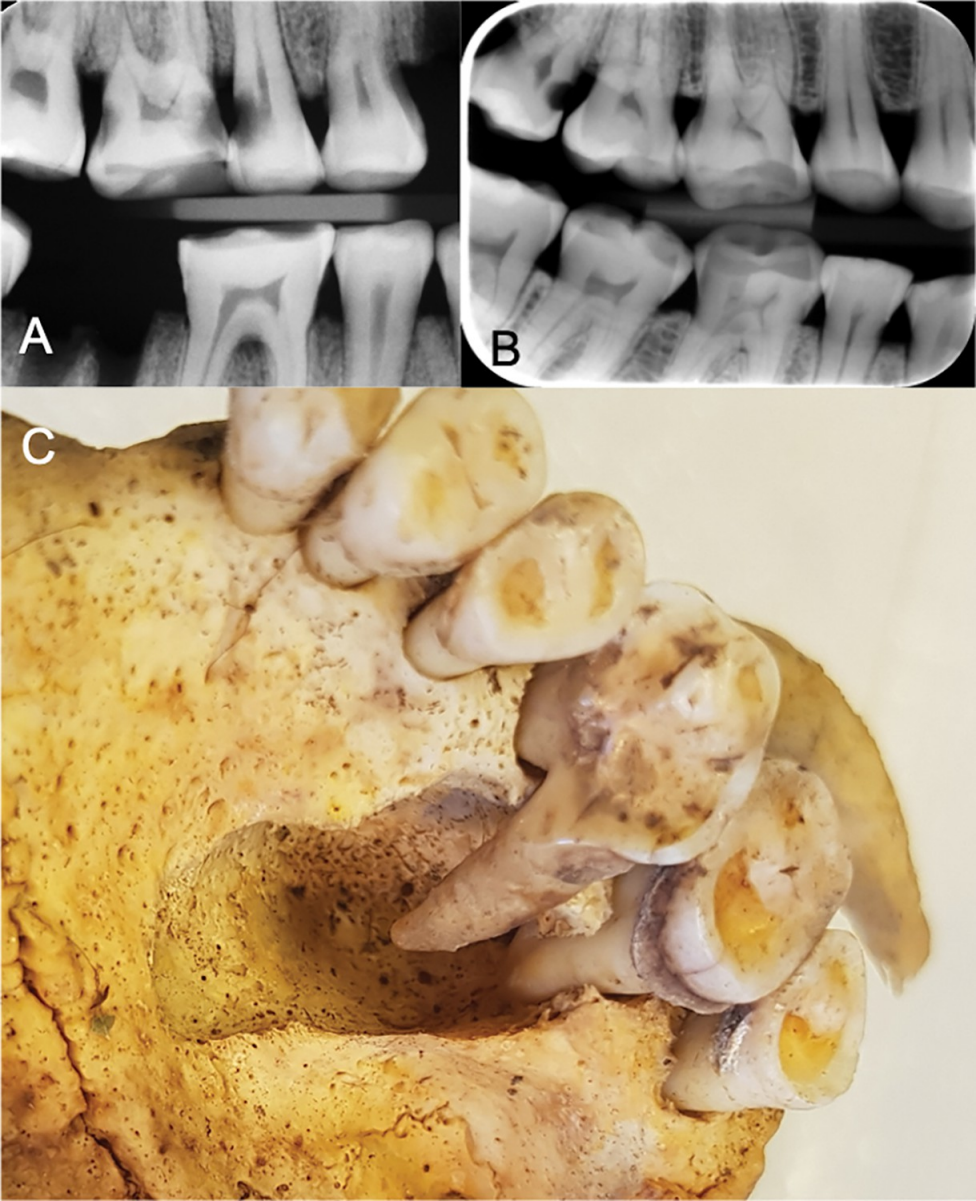
Dental pathological conditions. A) Manifest carious lesions in the first maxillary molar and second maxillary bicuspid, B) Manifest carious lesions in the second and third maxillary molars and C) Periapical lesion on molar 26 spreading medially in the palate. Possibly a cyst.

Almost half the population, 83 of 171 individuals (49%), had at least one carious lesion. All juvenile individuals were caries-free. In adults, at least one carious lesion was found in 62% of the population, while 38% were caries-free. Including missing teeth, 71% had DMT > 0, while 29% had DMT = 0. The mean DMT was 4.4. Of the 3,293 examined permanent teeth, a total of 13% displayed at least one carious lesion. The distribution of carious lesions was as follows: 7% of the teeth had crown caries, 7% root caries and 1% of the teeth displayed caries in both crown and root. The location most susceptible to caries was the root surface (25%), followed by the occlusal (19%), mesial and distal (15%) and the lingual/palatal surfaces (13%). The range in the number of carious lesions per individual varied from 0 to 22 (see Fig 2). A negative correlation was found (p<0.05) for the number of teeth with caries and age (see Fig 1). The number of carious, and lost teeth (post- and ante mortem) is presented in Fig 3. For proximal manifest carious lesions, 100% conformity was found between clinical and radiographic diagnostics. Other sites were not assessed on x-rays. On the proximal surfaces, only a few initial approximal carious lesions were found during radiographic screening and none was found during the clinical examination. Illustrations of dental caries are shown in Fig 4A and 4B.

### Other findings

A total of 4% of the examined teeth displayed apical lesions detectable clinically. The mean dental wear for molars was 3.0, while the mean dental wear for incisors was 2.8.

**Fig 5:**
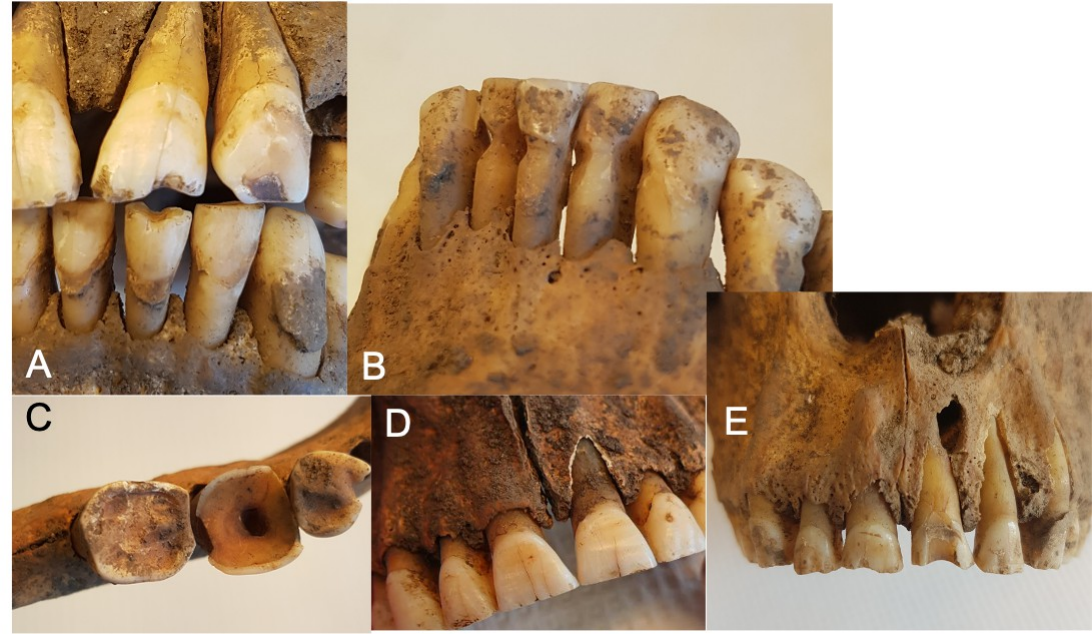
Manipulated teeth. A) Signs of habitual wear of the central incisors, B) Signs of tooth picking in the lower incisors, C) Signs of modification with the opening of the pulpal chamber of a mandibular molar, D) Filed front teeth and E) Modified central incisor with fistulated apical infection.

One male individual had characteristically filed front teeth (Fig 5). A total of five individuals displayed aplasia of teeth, of which four individuals were missing tooth number 12 or 22 and one individual both 45 and 35. One individual had 2 peg-shaped laterals and two individuals had a non-erupted canine in the maxillae. In two individuals, a molar (46 in both cases) appeared to have been modified to create an opening to the pulpal chamber. One individual showed modification in the buccal enamel to an incisor with apical periodontitis. Seven of the individuals displayed signs of atypical, habit-related tooth wear.

**Fig 6:**
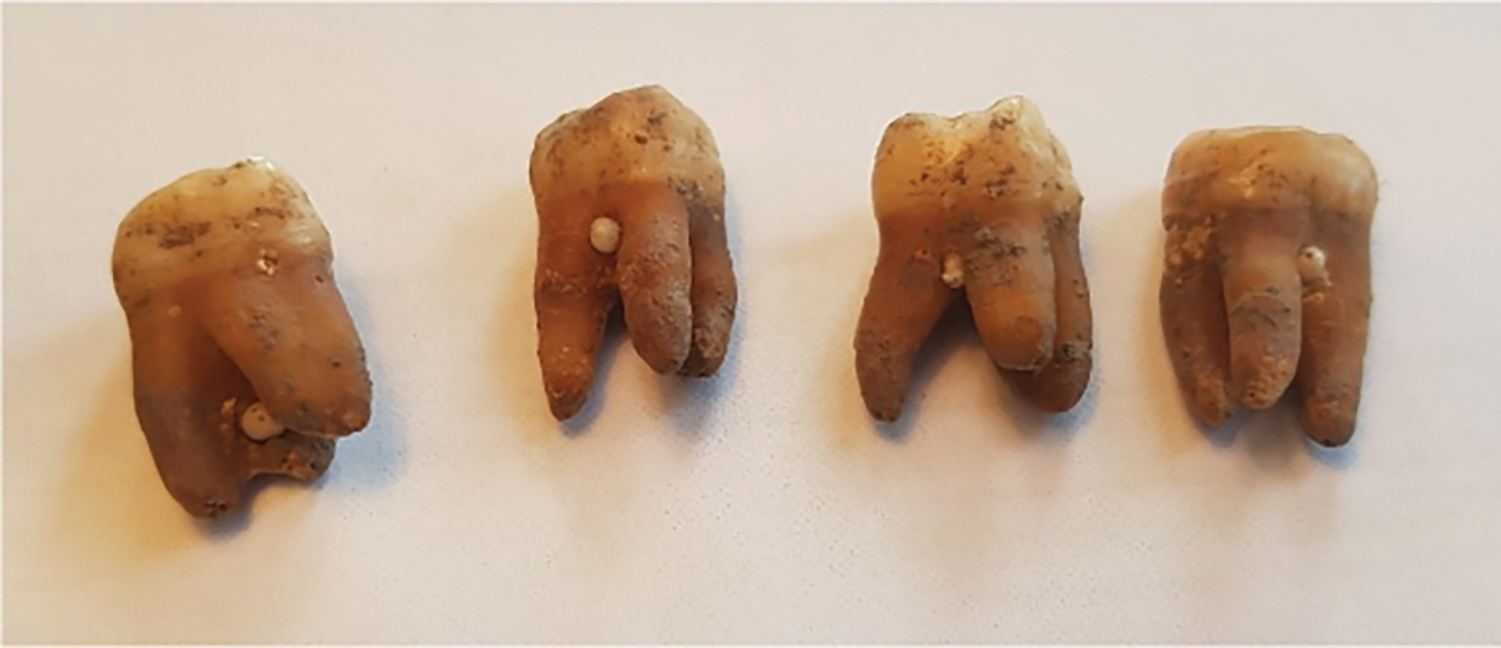
Anatomical variations. Molars with enamel pearls.

Two individuals displayed shortened roots of one or more teeth, while another individual exhibited resorption on a second molar from a wisdom tooth in the lower jaw. Enamel pearls, all located in molars, were discovered in four individuals (Fig 6). In general, the dentitions were neutral, with harmonizing relationships, while the crowding of teeth, as well as malocclusions, were rare. However, one individual was discovered with complete negative overjet i.e. underbite (mesio-occlusion).

## Discussion

The findings in this study provide rare insights into the Varnhem Vikings, at both the individual and the group level. The caries prevalence in the studied population correlates well with findings from other European populations from the same time period [9]. Interestingly, the surface that was most susceptible to dental caries was the root surface (25%). In modern-day populations, the prevalence of root carious lesions is growing with increasing biological age and it is more common in elderly [26]. This is explained by the exposure, in comparison to enamel, of the less mineralized root surface due to recession of the gums over time. The root surface is a well-known predilection site for carious lesion formation, due to an increased retention of the bacterial biofilm [26]. Gum recession could occur due to the loss of marginal bone in periodontal disease or in some cases abrasive brushing. The high occurrence of root caries in the studied Viking population could be linked to periodontal disease, of which signs in the form of marginal bone loss could be seen clinically and in the bitewings. It is probable that no oral hygiene measures other than tooth picking were implemented, thus causing the biofilm to remain on the root surfaces for longer periods of time. Notably, at the sites with abrasion marks from tooth picking, no carious lesions were found. A small part of the population displayed extraordinary numbers of carious lesions, while the majority were caries-free or displayed one or a couple of lesions. This polarization of caries prevalence is highly interesting, since it can also be seen in contemporary populations [27] and implies that some individuals have greater susceptibility to the caries disease. In this study, the majority of large cavitated lesions easily detected by clinical examination, large numbers of lost teeth and only a few initial carious lesions exclusively detected on x-rays provide an understanding of the nature of the caries disease when left untreated and without the intervention of modern-day dentistry.

The large number of lost teeth in this population, particularly the post-mortem loss, must be regarded as a source of error when assessing caries prevalence. Since this problem is widely recognized in the study of dental anthropology, attempts have been made in the literature to make models to correct for the bias of lost teeth when assessing the caries prevalence in archeological populations [28].

Due to the uneven distribution between males (n = 87) and females (n = 46) in the study population, it was not possible to draw any conclusions regarding sex differences in caries prevalence and tooth loss. This discrepancy between the sexes is explained by the excavation site, which is located mainly south of the church due to the topography of the area. According to early Christian customs, men were buried on the south side of the churches, and women in the north [29].

It was, however, possible to study correlations regarding age, tooth loss, and caries prevalence. The positive correlation found in the current study between biological age and ante-mortem tooth loss was not surprising and similar results have been reported in other historical populations [10, 12, 15, 30, 31]. It is plausible that the problem of dental caries contributed to the tooth loss, but other factors including periodontitis and trauma must be considered [2]. However, the ante-mortem tooth loss is thought to predominantly caused due to caries and extraction, since the caries prevalence was high in the remaining teeth. When studying the occurrence of untreated dental caries in a population, the prevalence might be expected to be commensurate with age, however, in this population, a decreasing caries rate was correlated with increasing age. This phenomenon can, when combining the factors of ante-mortem tooth loss and the occurrence of dental caries in current population, be explained by the decreasing number of teeth remaining with increasing biological age. Interestingly, by around the 40th year of life, the loss of teeth overrides the occurrence of carious lesions in the studied Vikings. This provides insights not only into the sufferings of the Varnhem Vikings but also into the pathology of dental caries in its untreated form.

Most Swedish Vikings, including the Varnhem population, lived in farm-based communities [32]. The diet in these communities was highly seasonal and included meats such as beef, pork and mutton [33]. Fish was consumed, as well as dairy products from the farm animals, bread and porridge and vegetables, such as pulses (grey beans and broad beans), cabbage, turnips and leeks [34]. The Vikings also ate hazelnuts and mushrooms. Fermentable carbohydrates came from only three natural sources: fruits or berries, honey, and malt [33]. The most essential drink was beer, which was available to every stratum of society, but milk and mead were also consumed. Even though wells were used during the Viking Age, the cleanliness of the water must have been insufficient for everyday drinking without processing [33, 34]. The carbohydrate components of the diet consisted of barley, wheat, oats, rye and peas, which were prepared to make bread, porridge and soup [32]. The coarseness of the food contributed to the wear seen in the teeth of those at Varnhem and other contemporary populations [32]. The high intake of starchy foods, in combination with the lack of dental care and hygiene, and sites with food impaction between teeth partly explain the occurrence of caries disease in this population [35]. It is impossible to know whether the cohort acquired and consumed additional cariogenic victuals uncommon to this era and location. However, due to the multifactorial etiology of the disease, it is important to consider other, for this population unknown, modifying factors [36] relating to saliva, microflora, hygiene, genetics, culture and physiology. Environmental factors such as fluoride in drinking water may also have had an impact on caries prevalence.

The validation of clinical examinations with radiographic imaging in this work indicated that, clinical examination is a valid method for detecting manifest carious lesions. However, additional initial lesions would probably have been found with extensive radiographic imaging, which has been proven in the literature [37]. Nevertheless, the fragmented state of many of the individual remains prohibited the use of radiographic imaging and bitewings for the entire studied population. Due to the nature of the disease during this time period, it is believed that most lesions, with no or only a few caries-prevention strategies available, quickly progressed once a lesion was established.

Some of the most important findings in this study are those indicating infections. Clinically visible signs of dental infections were seen in 4% of the teeth. In the palate of one individual, a 30- to 35-year-old woman, bone resorption due to an extensive infection was seen in proximity to the palatal roots of teeth 26 and 27 (Fig 4C). This infection would have been hazardous during the time period with no available treatments. It may very well be possible that the infection spreading through the soft tissues could have caused death, due to either obstruction of the airways, or sepsis.

Large carious lesions seen clinically and radiographically indicate that several of the studied Vikings suffered from tooth pain, since the cavities had reached close proximity to the pulp (Fig 4B). It is also probable that food fragments were stuck in the open carious sites, causing food impaction and discomfort (Fig 4A). In several individuals, abrasion like that made by toothpicks (Fig 5B) could be seen, indicating a habit of removing food debris from interproximal areas. Similar wear has been found in other Viking Age dentitions [21, 32] and also in populations as early as Neanderthals [38]. Other traces of tooth manipulation were found, for instance, there were two cases where findings on the mandibular first molars indicated that an opening and widening of the pulpal chamber had been created, most likely in an attempt to relieve pain (Fig 5C). Another case showed heavy abrasion to a maxillary incisor with an apical infection (Fig 5E). These are interesting and important findings that indicate that the Vikings in this population performed more complex interventions regarding dental diseases than the mere extraction of hurting teeth. There are several archaeological examples of prehistoric dentistry such as dental fillings from the Neolithic Period in Slovenia [39] that had been performed using beeswax, as well as drilling of teeth in Neolithic Pakistan [40]. However, this is the first time that signs of treatment of dental pathological conditions have been found in Swedish Viking remains.

Interestingly, one individual in this population displayed filed front teeth (Fig 5D), the same type of modification seen in other, mainly Swedish, Viking Age individuals [32, 41, 42]. This individual was male, 35–50 years, and had, in addition to the filed teeth, heavily worn molars and three proximal caries lesions located in the premolar and molar of the right maxillae. The filed marks consist of horizontal lines in the dental enamel of the central and lateral incisors. The reason as to why some of the Vikings filed their teeth is unknown, however, it is suggested to have been a marker of identity [32]. In the literature, all the identified Viking individuals with filed teeth are male and many of these remains display skeletal injuries from weapons [41]. Geographically, the most common site with individuals exhibiting this kind of dental modification is on the Swedish island of Gotland, but cases have been reported from other locations in Sweden. Additionally, solitary findings have been reported in England and Denmark and, interestingly enough, in one individual found in Egypt [41].

In conclusion, the findings in this present study provide rare insights into early Christian life in a Swedish Viking settlement. A relatively high prevalence of carious lesions, in both teeth and individuals, indicates nutritional components of digestible carbohydrates such as starches. Tooth pain and tooth loss, as a result of dental caries, attrition and infections, were common and affected everyday life. The findings included potential life-threatening oral infections and indications of the habitual usage of toothpicks and modifications of teeth, in order both to change appearances and perhaps to also relieve pain. These findings provide an extraordinary glimpse of a long-lost world and a rare and essential understanding of our ancestors, and life and death during the Viking Age.

## Supporting information

S1 Table Samples, description and raw data. This table contains the identity, biological markers and pathological conditions of the studied population.
(XLSX)
